# The role of metabolic factors in the association between obesity and cholelithiasis: A two-step, two-sample multivariable mendelian randomization study

**DOI:** 10.1016/j.clinsp.2024.100520

**Published:** 2024-10-19

**Authors:** Xiangrong Xu, Jiawei Gao, Jun Sun, Ruiwen Liu, Wei Chen

**Affiliations:** a Department of General Surgery, Kunshan Hospital Affiliated to Jiangsu University, Kunshan, PR China; b Department of General Surgery, Second Affiliated Hospital of Soochow University, Suzhou, PR China

**Keywords:** Body mass index (BMI), Metabolic factors, Mediation, Cholelithiasis, Mendelian randomization study

## Abstract

**Background and purpose:**

The extent to which the effects of BMI on cholelithiasis are mediated by metabolic factors (including blood pressure, blood lipids, body mass, and fasting blood glucose) is unclear. Therefore, in this study, the authors used genetic evidence to test the effects of these characteristics.

**Methods:**

Summary-level data for exposures and main outcomes were extracted from GWAS consortia. The authors used a two-step, two-sample Multivariable Mendelian Randomization (MVMR) analysis to illustrate the effect of BMI on cholelithiasis and a stepwise test method to quantify the possible mediating effects of cardiometabolic factors on cholelithiasis.

**Results:**

For each one-unit logarithmic increase in body mass index, the risk of cholelithiasis increased by 98 % (Odds Ratio [OR = 1.98], 95 % CI: 1.73 %‒2.28 %). After mediation analysis, the authors found that high-density lipoprotein and triglycerides were the main mediating factors, while the mediating effects of other metabolic factors were not significant. The total effect ratios of HDL and TG on cholelithiasis were 7.3 % (95 % CI: 8.51 %‒12.85 %) and 3.5 % (95 % CI: 3.59 %‒6.50 %), respectively. HDL and TG played a significant role in regulating cholelithiasis, but there was no evidence to show the regulatory effect of LDL on cholelithiasis. The total effects of BMI and triglycerides on cholelithiasis were 10.7 % and 5.0 %, respectively.

**Conclusion:**

The authors found that among the metabolic factors evaluated, the decrease of HDL and the increase of TG mediated a high proportion of the effect of BMI on cholelithiasis. Therefore, intervention with these factors may reduce the increased risk of cholelithiasis in patients with high BMI.

## Highlights

• Genetic analysis shows HDL mediates 7.3 % of BMI's impact on cholelithiasis risk.
• Triglycerides contribute 3.5 % to BMI's effect on cholelithiasis, according to genetic evidence.
• Targeting HDL & Triglycerides may reduce cholelithiasis risk in individuals with high BMI.
• Study reveals novel insights into BMI-associated cholelithiasis mechanisms.

## Introduction

Cholelithiasis, a prevalent biliary disease, is primarily caused by gallstone obstruction of the cystic duct, leading to bile stasis and inflammation.^1^ Elderly patients are particularly vulnerable, with an increasing incidence of acute cholelithiasis accompanied by a rise in complications, admissions, readmissions, and mortality.^2^^,^^3^ Therefore, early detection of patients with cholelithiasis and effective intervention can greatly improve the prognosis of patients.

Recently, the increase in the incidence of cholelithiasis coincided with the global epidemic of obesity, which suggests that higher BMI may be an independent, potential and preventable risk factor for cholelithiasis.^4^ The imbalance between pro-inflammatory and anti-inflammatory responses induced by obesity is one of the mechanisms for the deterioration of cholelithiasis.^5^ Second, obese people are prone to developing chronic diseases closely related to poor lifestyles. Large-scale prospective population studies have shown that a higher BMI is associated with poor blood lipid levels, higher fasting blood glucose, and hypertension.^6^ Randomized controlled trials^7^ have shown that elevated triglycerides, low-density lipoprotein, cholesterol, glucose, and blood pressure increase the risk of cholelithiasis. Therefore, the association between BMI and cholelithiasis may be mediated by these identified modifiable risk factors. Based on this, exploring treatment strategies other than weight control is very important for the management of patients with high BMI. Understanding whether these risk factors play a role in the effect of BMI on cholelithiasis will provide new intervention targets for patients with high BMI to reduce the excessive risk of cholelithiasis.

However, due to unknown or unmeasured confounding factors, the most frequently used observational studies have been criticized for their weakness in proving causality. In addition, a well-designed Randomized Controlled Trial (RCT) is time-consuming and expensive, and it requires consideration of important ethical factors. Therefore, whether this intermediary effect is causal is still largely unknown.

Mendelian Randomization (MR) is a genetic epidemiological method that uses genetic variation as an instrumental variable to assess the potential associations between risk factors and outcomes.^8^ MR is widely used to study the potential effects of exposure on outcomes.^9^ In the two-sample MR method, the genetic variation of exposure and outcomes are extracted from different datasets, which increases statistical validity. Therefore, over the past decade, MR has increasingly been used to provide more reliable estimates of the effects of many risk factors on a range of health outcomes, and the results of MR are very similar to those of randomized controlled trials (such as those on the effects of HDL^10^ and TG^11^ on cholelithiasis) due to the rapid increase in genetic research and genomics and the widespread use of bioinformatics. Now, a method has been developed to test mediation using a two-step method, which greatly reduces the inherent limitations of common multivariable methods.^12^

Previous MR studies have shown the potential effects of BMI and metabolic factors on cholelithiasis,^13^^,^^14^ but none of them have quantified the mediating effects of those factors. Therefore, to understand how much of the effect of BMI on cholelithiasis is mediated by metabolic factors, the authors conducted a two-step, two-sample multivariable MR study. The purpose of this study was to assess the mediating effects of metabolic factors, including fasting blood glucose, hypertension, LDL, HDL, and TG, on cholelithiasis by analyzing data from a Genome-Wide Association Study (GWAS) from the International Genetic Alliance (IGA).

## Methods

### Overall study design

The two-step, two-sample Mendelian Randomization (MR) study utilized publicly available datasets to investigate the genome-wide associations between exposure and outcome factors. The two-sample MR approach allows us to estimate exposure-variant associations in one dataset and outcome-variant associations in a separate dataset.

First, the authors tested the effects of BMI on cholelithiasis using genetic variants associated with BMI from one dataset as instrumental variables. Then, to examine the potential mediating role of cardiovascular metabolic factors, the authors performed a two-step MR analysis.

In step one, the authors utilized the first dataset to examine the associations between BMI-associated genetic variants and potential mediators.

In step two, the authors leveraged a second, independent dataset to investigate and quantify the potential mediating role of these cardiovascular metabolic factors in the relationship between BMI-associated genetic variants and cholelithiasis. By analyzing these two separate datasets, the authors were able to assess the mediating effects while minimizing potential biases that may arise from using a single dataset.

### Data sources

#### Genetic instrumental variables for BMI

From the latest genome-wide association study of BMI available on the GWAS website (https://gwas.mrcieu.ac.uk/datasets/ukb-b-19953/), the authors identified 458 SNPs (*p* < 5 × 10^–8^) that independently contributed to BMI in a primary meta-analysis of genetic variation of more than 12 million individuals of European ancestry. Based on the low correlation (R^2^ < 0.001) in HapMap22 or 1000 Genome Project data, these variants are defined as independent of each other.

#### Genetic instrumental variables for potential mediators

A total of five metabolic factors, including blood pressure, blood lipids, and fasting blood glucose, were selected as potential mediators.

For these potential intermediaries, the authors obtained data from the online public GWASs of participants of European descent provided by UK Biobank and MRC-IEU (http://Gwas-api.mrcieu.ac.uk/) using the ‘TwoSampleMR’ package of R software version 4.3.1 (R Consortium, Boston, Massachusetts). The authors obtained genetic variations in high-density lipoprotein, low-density lipoprotein, and total cholesterol from 441,016 European participants. For fasting blood glucose and hypertension, SNPs were extracted from GWASs, which included 13,556 and 463,010 European participants, respectively.

### Data on cholelithiasis

The authors extracted genetic variation data for cholelithiasis from the UK Biobank Consortium (https://gwas.mrcieu.ac.uk/datasets/ieu-b-4971/). The summary-level statistics are from a large study involving 4052 cases and 48,232 controls, all of European ancestry. In this large study, cholelithiasis was diagnosed based on the International Classification of Diseases, 10th Revision (ICD-10) and code K80 definitions.

### Statistical analysis

#### Effect of BMI on cholelithiasis

The effects of BMI on cholelithiasis were estimated using a two-sample MR analysis.^15^ As an indication of the strength of the association between genetic instruments and phenotypes, the authors report the proportion of variation in BMI and all mediators explained by their genetic variant instruments. The proportion of the BMI–Cholelithiasis effect that is explained by a group of mediators will be estimated with bias if the mediators are related to each other and/or if the outcome has an effect on the mediator (i.e., there is reverse causality), and the instrument affects the mediators through the outcome. Therefore, the authors tested for potential bidirectional effects of BMI, potential mediators, and cholelithiasis with each other using the Inverse-Variance Weighting (IVW) approach described below. The results are shown as Odds Ratios (ORs) and 95 % Confidence Intervals.

#### Effects of BMI on metabolic factors

Similarly, the authors used two-sample MR to estimate the effect of BMI on each cardiometabolic factor. The results were expressed as the β coefficients and 95 % CIs. Since some cardiometabolic factors are derived from the same database, it is important to use Bonferroni correction thresholds, where *p* < 0.05 is considered a potential association and *p* > 0.05 indicates no significant relationship with BMI.

#### Effects of metabolic factors on cholelithiasis

Then, the effects of each metabolic factor on cholelithiasis were evaluated again using two-sample MR methods. The results show the use of OR and 95 % CI; *p* < 0.05 was considered indicative of a potential association. Cardiometabolic factors that did not meet the *p* < 0.05 criteria were excluded.

#### Mediation effects of metabolic factors

To calculate the mediating effects of cardiometabolic factors, the estimated effects of BMI on metabolic factors were multiplied by the estimated effects of cardiometabolic factors on cholelithiasis to obtain the individual mediating effects of each cardiometabolic factor. The authors then divided the mediating effect by the total effect of BMI on cholelithiasis to obtain the mediating ratio of each mediating agent, thus obtaining the possible effect of BMI on cholelithiasis.

### Sensitivity analyses

The authors used Inverse-Variance Weighting (IVW) methods to examine potential effects between BMI, potential mediators, and cholelithiasis. Horizontal pleiotropy, in which genetic variants influence outcomes through pathways other than exposure, violates MR's assumptions and may lead to bias in estimates. To prevent this from happening, the authors used a two-step approach, in which five different analytical methods were used in the first step (effect of BMI on Cholelithiasis and potential mediators) and the second step (effect of potential mediators on Cholelithiasis). Each of the five approaches assumes a different model of horizontal pleiotropy. The value of comparing the results of these five methods is that the authors can be more confident of consistent results between the different methods.

To estimate the effect of BMI on cholelithiasis, taking into account the potential mediators determined by genetics, the authors used the IVW method and adjusted the potential mediation effect of each SNP.^16^ The proportion of the effect mediated by any underlying mediator is estimated by the change in the total effect of genetically determined BMI on cholelithiasis risk, which assumes that the mediator is a continuously measured variable.

R version 4.3.1 (the R Foundation for Statistical Computing, Vienna, Austria), including the ‘TwoSampleMR’ and ‘MendelianRandomization’ packages, was used for all of the above analyses. The calculation rules of Se are derived from the normal distribution error of the Gaussian distribution equation suitable for different cases, such as addition, subtraction, multiplication, and division.

## Results

The present study conducted a primary meta-analysis of genetic variation in over 12 million individuals of European ancestry to identify SNPs associated with BMI. Specifically, a total of 458 SNPs (*p* < 5 × 10^–8^) were found to independently contribute to BMI. In addition, the authors analyzed genetic variations in high-density lipoprotein, low-density lipoprotein, and total cholesterol among a subset of 441,016 European participants. For fasting blood glucose and hypertension, SNPs were extracted from separate Genome-Wide Association Studies (GWASs), which comprised 13,556 and 463,010 European participants, respectively.

The study population had an average age of 48 years, ranging from 19 to 82 years. The gender distribution was approximately 58 % male and 42 % female, reflecting a broad representation of the European ancestry population.

### Selected SNPs for BMI

After excluding SNPs that did not meet the criteria for genome-wide significance (*p* < 5 × 10^–8^) and clusters of SNPs with linkage disequilibrium (r^2^ < 0.001), this study ultimately selected 458 SNPs associated with BMI. Furthermore, these SNPs have F statistics greater than 10.

### Total effect of BMI on cholelithiasis

**Fig. 1:**
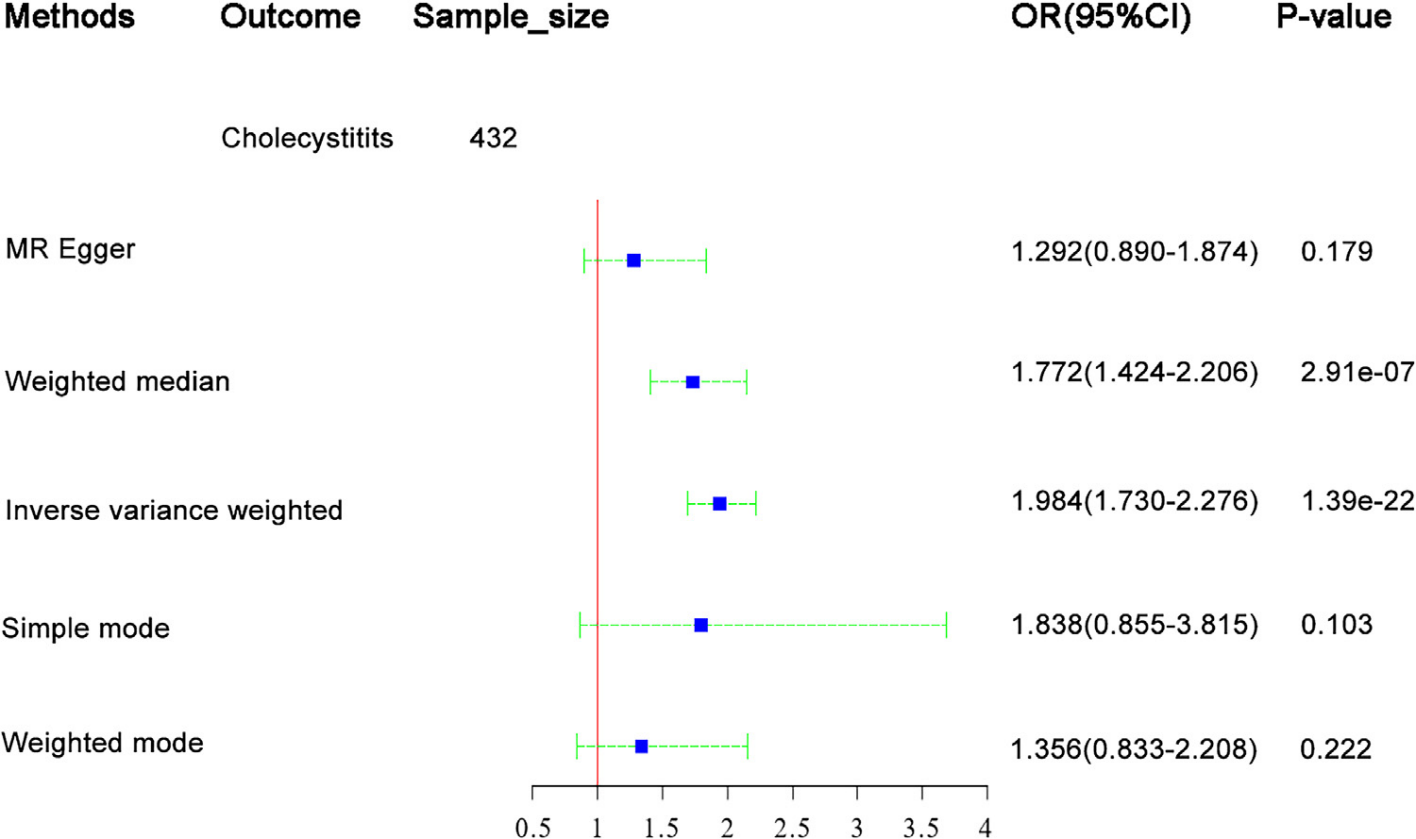
Estimates of casual effect of body mass index (BMI) on cholelithiasis in MR analysis.

The present study showed the total effect of BMI on cholelithiasis (Fig. 1). One unit logarithmic in BMI increased the risk of cholelithiasis by 98 % (OR = 1.98, 95 % CI 1.73‒2.28, *p* < 0.001).

### Effect of BMI on metabolic factors

**Fig. 2:**
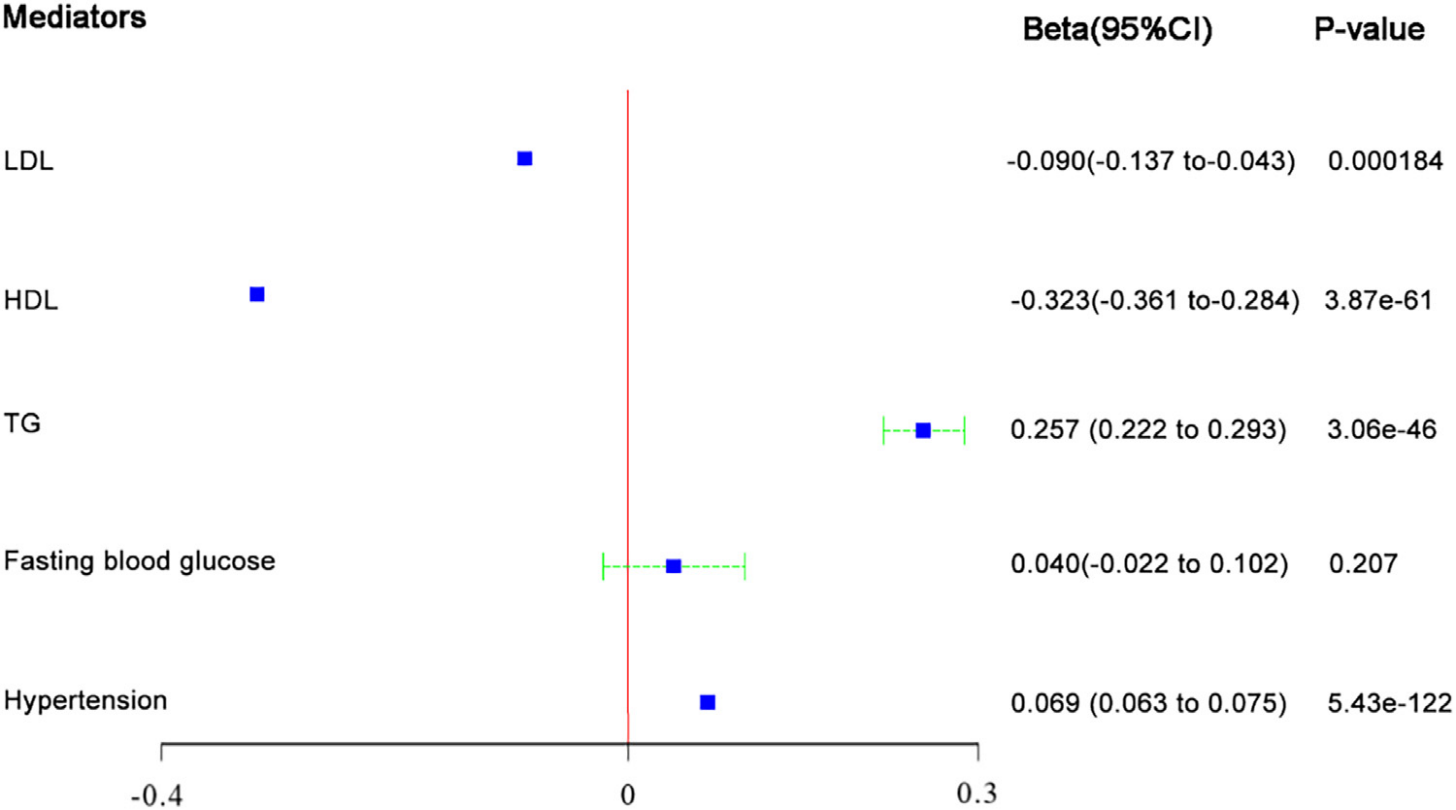
Estimates of casual effect of body mass index (BMI) on metabolic factors in MR analysis.

Fig. 2 shows a one-unit logarithmic increase in BMI is associated with a one-SD increase in TG (β = 0.26, 95 % CI: 0.22‒0.29, *p* < 0.001) and hypertension (β = 0.04, 95 % CI: 0.06‒0.08, *p* < 0.001) and a one-SD decrease in LDL (β = −0.09, 95 % CI: −0.14; −0.04, *p* < 0.001) and HDL (β = −0.32, 95 % CI: −0.36; −0.28, *p* < 0.001). A Bonferroni corrected p-value threshold was used to indicate statistical significance for those analyses. No effect of BMI on fasting blood glucose (*p* = 0.21) was found.

### Effects of metabolic factors on cholelithiasis

**Fig. 3:**
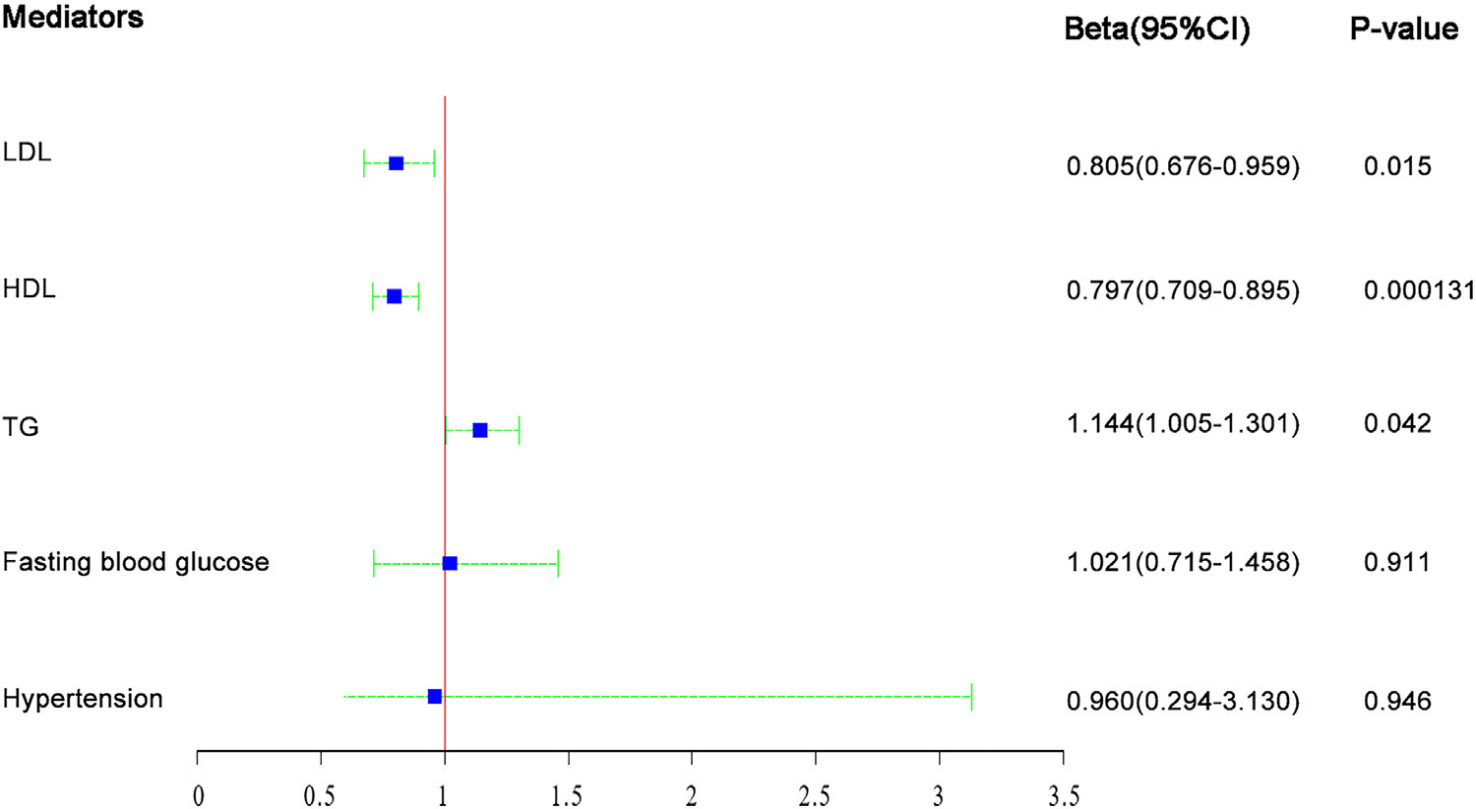
Estimates of casual effect of metabolic factors on cholelithiasis in MR analysis.

Fig. 3 shows the estimate of the effect of a one-SD increase in each metabolic factor on cholelithiasis after adjusting for BMI. The estimated log odds of cholelithiasis for a one-SD increase in LDL, HDL, TG, fasting blood glucose and hypertension were 0.81 (95 % CI: 0.68‒0.96, *p* = 0.015), 0.80 (95 % CI: 0.71‒0.90, *p* < 0.001, 1.14 (95 % CI: 1.00‒1.32, *p* < 0.001), 1.02 (95 % CI: 0.72‒1.46, *p* = 0.911), and 0.96 (95 % CI: 0.29‒3.13, *p* = 0.946), respectively.

### The mediating role of metabolic factors in cholelithiasis

After excluding the metabolic factors that were not causally influenced by BMI and those that did not demonstrate a clear association with cholelithiasis, the authors chose LDL, HDL, and TG for mediation analysis.

Table 1 shows the proportion of effects of BMI on cholelithiasis mediated by each metabolic factor included in the intermediary analysis. Among the effects of BMI on cholelithiasis, the proportions mediated by LDL, HDL, and TG were 2.8 % (0.003 %‒5.7 %), 10.7 % (8.5 %‒12.9 %) and 5.0 % (3.6 %‒6.5 %), respectively.

**Table 1:** Mediation effect of BMI on cholelithiasis via metabolic factors.

	Mediation effect (%)	Mediation/Total effect (%)	95 % CI	p
**LDL**	1.95	2.84	0.003; 5.673	0.317
**HDL**	7.32	10.68	8.511; 12.852	<0.001
**TG**	3.46	5.05	3.588; 6.505	<0.001

## Discussion

In this large-scale multivariable MR study, the authors estimated that each one-unit logarithmic increase in BMI increased the risk of cholelithiasis by 98 %. More importantly, the authors found that blood lipids played an important mediating role in the risk of cholelithiasis in patients with high BMI. In particular, HDL, as an important regulator, accounted for 7.3 % of the additional risk of cholelithiasis. Therefore, appropriate interventions for these mediating factors may reduce the risk of cholelithiasis in most patients with high BMI.

BMI, an indicator of general obesity, has been reported to be causally associated with an increased risk of cholelithiasis by two MR studies.^17^^,^^18^ However, BMI is a comprehensive index with varying applicability to diverse populations, and numerous factors, including eating habits and body composition, contribute to fluctuations in BMI. Consequently, it is impracticable to precisely forecast the risk of cholelithiasis based solely on BMI. Prior to this, several studies have confirmed that a high BMI is associated with a high risk of cholelithiasis.^19^^,^^20^ Studies such as the one by Littlefield have shown that the 10-year cumulative risk of cholelithiasis in obese people (BMI ≥28.0 kg/m^2^) is 18.4 %, while the 10-year cumulative risk of cholelithiasis in overweight people (BMI ≥ 24.0 kg/m^2^) is 10.9 %.^21^ This is consistent with the present results, but observational studies are affected by many other confounding factors and measurement errors, such as whether patients in the study have risk factors associated with cholelithiasis such as diabetes, hyperlipidemia, or obesity predisposition.^22^ Compared with observational studies, the present results are more consistent with previous MR studies. One MR study showed that a one-unit logarithmic increase in BMI was associated with an increased risk of cholelithiasis (OR = 1.63, 95 % CI: 1.36‒1.96), abnormal LDL (OR = 0.79, 95 % CI: 0.70‒0.90) and abnormal HDL (OR = 0.95, 95 % CI: 0.85‒1.06). However, that part of the study did not further explore the possible intermediary effects.^23^

This study shows that the decrease in HDL and the increase of TG are the main mediators of cholelithiasis in patients with high BMI. Previously, many observational studies have suggested that BMI is associated with HDL and Triglycerides (TG).^24^^,^^25^ However, few studies have investigated whether these metabolic factors play an intermediary role in the risk of cholelithiasis in patients with BMI.^26^ The present study further revealed that the mediating effects of the decrease of HDL and the increase of TG accounted for 10.7 % and 5.0 % of the total effects, respectively. It is suggested that clinical intervention on these factors may help to reduce the risk of cholelithiasis in patients with high BMI. At present, metabolic factors are still controversial as treatment targets for the risk of cholelithiasis in patients with high BMI. Due to limitations of the study design, sample size, or other factors, there is still not enough evidence to show that drugs that reduce TG or increase HDL can reduce the risk of cholelithiasis in patients with high BMI. These results demonstrate a need for new large-scale randomized controlled trials to investigate whether genes related to TG and HDL can be new therapeutic targets for reducing the risk of cholelithiasis in patients with high BMI.

At present, most of the pathophysiological mechanisms of cholelithiasis in patients with high BMI are considered to be related to leptin.^27^ Leptin is encoded by the obesity gene and is mainly secreted by white adipocytes. The secretion level peaks at night and subsequently declines rapidly, exhibiting a distinct circadian pattern. Certain research has revealed a notable difference in serum leptin levels between patients with cholelithiasis and healthy individuals. Nonetheless, this observation requires further validation through extensive clinical or animal studies.^28^ However, the pathophysiological mechanism of metabolic factors such as TG and HDL in cholelithiasis is still unclear. Some studies^29^ believe that high triglycerides can decrease the contractile function of the gallbladder, lead to poor gallbladder emptying, and then crystallize cholesterol in the gallbladder, leading to the occurrence of cholelithiasis. HDL^30^ plays a key role in reverse cholesterol transport, activating a variety of lipases and promoting lipid metabolism and decomposition, thus reducing the risk of cholelithiasis in patients with high BMI.

The present research has several advantages. This study is the first MR analysis of the mediating role of metabolic factors in the risk of cholelithiasis in patients with high BMI. In addition, the risk of reverse causality and confounding factors commonly seen in observational studies was reduced by using SNPs as variables for metabolic factors and MR methods. In addition, the included data sources for exposures and results come from the largest GWAS to date, with large sample sizes and reliable data. Moreover, the study is limited to the European population to reduce the bias caused by demographic stratification.

However, there are still some limitations in this study. First, due to the limited number of SNPs available, the analytical ability is relatively low. Second, because the GWAS data used are mainly based on the European population, the generalizability of these conclusions is limited, so it is necessary to verify these conclusions in other populations. Third, the use of genetic IVs shows that the effects of exposure to results are lifelong, which may be different from the actual situation. Finally, the study showed that the connection between high BMI and cholelithiasis was partly achieved through HDL and TG mediation. However, there may be other intermediary factors that the authors have not studied, which need to be further studied and explored.

## Conclusion

By using a two-step, two-sample MR analysis, this study presents compelling evidence for the association between BMI and the development of cholelithiasis. Furthermore, the results indicate that approximately 10.68 % of the additional risk of cholelithiasis in patients with high BMI is mediated by TG while 5.05 % is mediated by HDL. The authors believe that large-scale interventions targeting TG and HDL could reduce a substantial proportion of cholelithiasis risk among patients with high BMI.

## Ethics approval and consent to participate

The analyses were based on publicly available data that have been approved by relevant review boards. The UK Biobank was approved by the Research Ethics Committee (REC reference: 21/NW/0157). This article has followed the Strengthening the Reporting of Observational Studies in Epidemiology (STROBE) statement.

## Data availability

All the data were downloaded from https://gwas.mrcieu.ac.uk/datasets. All of these data are publicly available. Investigators who have made their genome-wide data available may not necessarily agree with comments made in this article and the authors take full responsibility for the content of this article.

## Authors’ contributions

JG, and XX are Data curation, Formal analysis. JG, XX, and JS are Writing – original draft. RL, and WC are Writing – review & editing. All the authors have read and approved the final manuscript.

## Funding

This research received no specific grant from any funding agency in the public, commercial, or not-for-profit sectors.

## Declaration of competing interest

The authors declare no conflicts of interest.
